# Supplementary material for: Direct observation of the formation and stabilization of metallic nanoparticles on carbon supports
Source: Nat Commun. 2020 Dec 11;11:6373. doi: 10.1038/s41467-020-20084-5 (PMC7733500; doi:10.1038/s41467-020-20084-5)
Supplement: Supplementary file 1 — Supplementaty Information [file 41467_2020_20084_MOESM1_ESM.pdf]

Supplementary Information for

Direct Observation of the Formation and Stabilization of Metallic Nanoparticles on Carbon Supports

Zhennan Huang[1a], Yonggang Yao[2a], Zhenqian Pang[3a], Yifei Yuan[1,4], Tangyuan Li[2], Kun He[5], Xiaobing Hu[5], Jian Cheng[3], Wentao Yao[6], Yuzi Liu[7], Anmin Nie[8], , Soroosh Sharifi-Asl[1], Meng Cheng[1], Boao Song[1], Khalil Amine[4], Jun Lu[4], Teng Li[3]*, Liangbing Hu[2]*, Reza Shahbazian-Yassar[1]*

1. Department of Mechanical and Industrial Engineering, University of Illinois at Chicago, Chicago, IL 60607, United States

2. Department of Materials Science and Engineering, University of Maryland, College Park, Maryland 20742, United States

3. Department of Mechanical Engineering, University of Maryland, College Park, Maryland 20742, United States

4. Chemical Sciences and Engineering Division, Argonne National Laboratory, Argonne, IL 60439, United States

5. Northwestern University Atomic and Nanoscale Characterization Experimental (NUANCE) Center, Northwestern University, Evanston, Illinois 60208, United States

6. Department of Mechanical Engineering-Engineering Mechanics, Michigan Technological University, Houghton, MI 49931, United States

7. Center for Nanoscale Materials, Argonne National Laboratory, Argonne, IL 60439, United States

8. Center for High Pressure Science, State Key Lab of Metastable Materials Science and Technology, Yanshan University, Qinhuangdao 066004, China

a. These authors contributed equally to this work

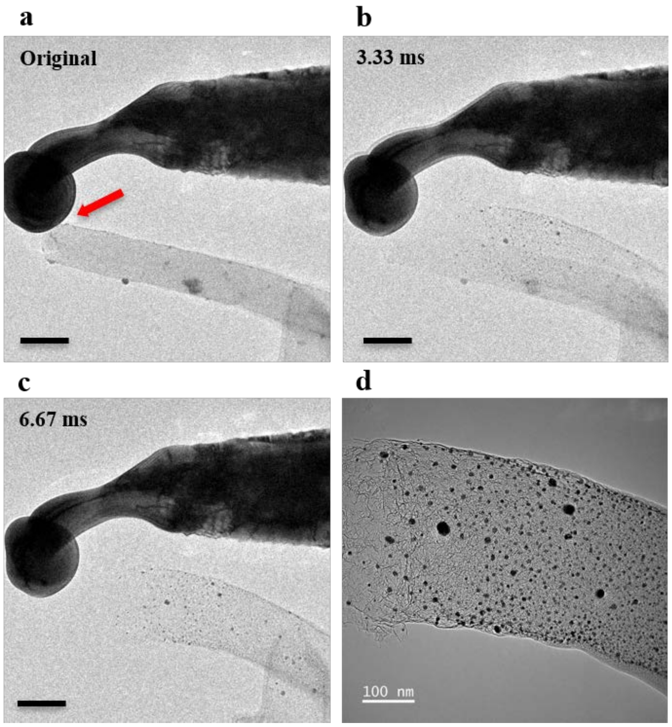

Supplementary Figure 1. In situ Joule heating of a salt loaded CNF (S-CNF) captured by ultrafast camera (300 fps) at low magnifications: (a) pristine status of S-CNF before the start of Joule heating; (b) TEM image taken after 3.33 ms showing the S-CNF expansion and the formation of Pt nanoparticles. The pristine S-CNF shadow image still appears in the background due to the camera recording being at the image capturing limit; (c) the TEM image of nanoparticles on expanded CNF captured after 6.67 ms; and (d) higher resolution TEM image of the Joule heated S-CNF with wrinkled structure and nanoparticles.

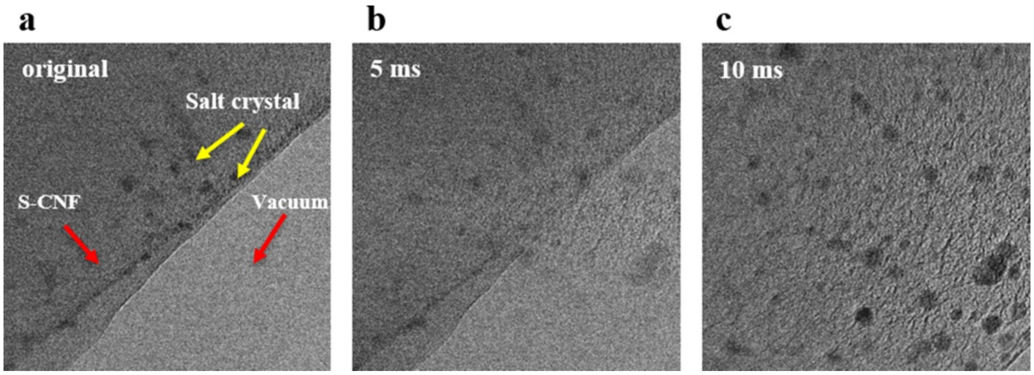

Supplementary Figure 2. In situ Joule heating of S-CNF captured by ultrafast camera (200 fps) at high magnifications. (a) Pristine S-CNF; (b) Joule heated S-CNF captured at 5ms showing particle formation and CNF expansion; (c) Joule heated S-CNF captured at 10ms showing the formed particles well associated with winkled structures.

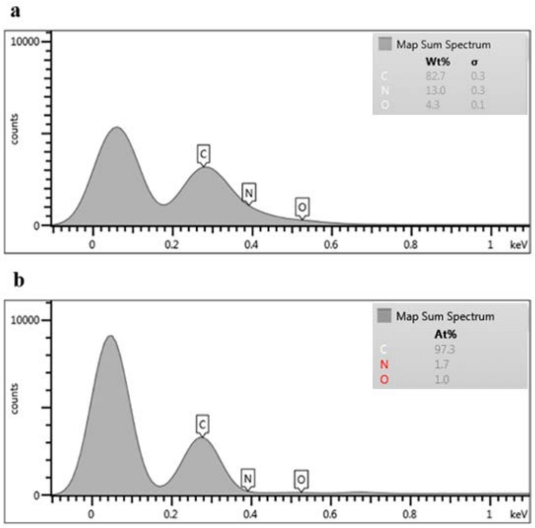

Supplementary Figure 3. EDS spectrum of CNF (a) before and (b) after Joule heating.

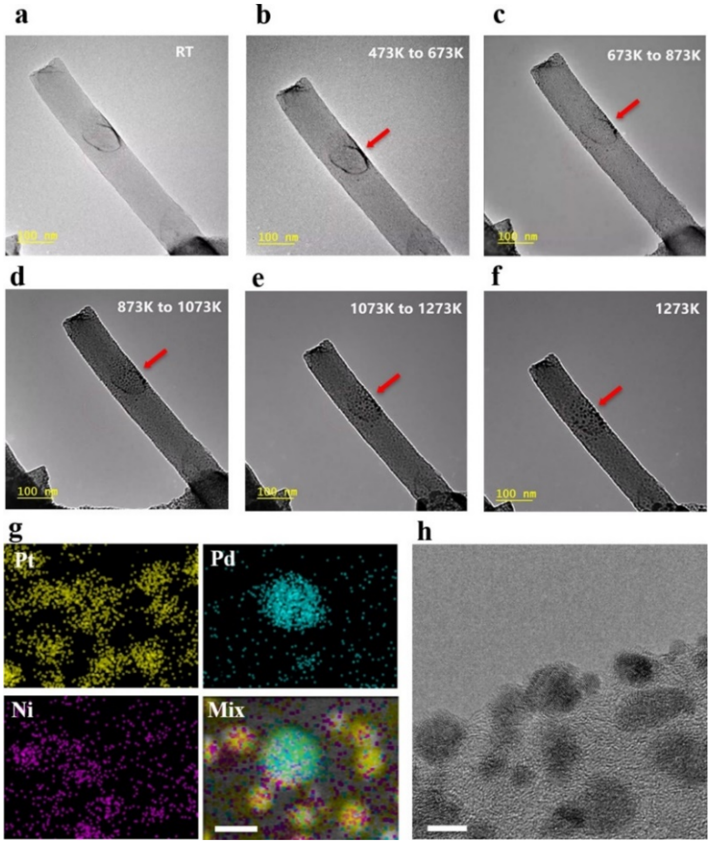

Supplementary Figure 4. In situ heating with SiN-based membrane TEM holders at heating rate of 200 K/s: (a-f) the formation of Pt nanoparticles on S-CNF marked by the red arrows; (g) the EDS maps show the elemental segregation of Pt, Pd and Ni after heating; and (h) high resolution TEM image show the smoothness of CNFs surface after the formation of PtPdNi nanoparticles. Scale bars are 5 nm.

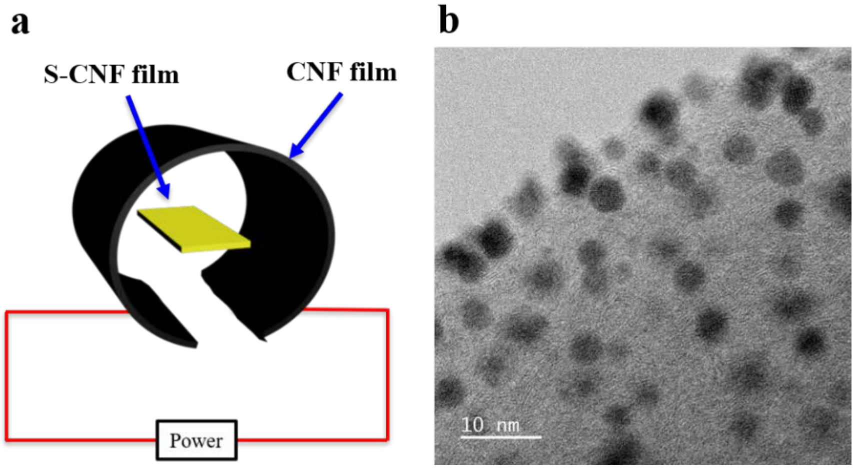

Supplementary Figure 5. (a) Schematic image of rapid radiative heating setup. A hollow cylinder made of CNF film was connected to electrodes on both ends with a large piece of salt coated CNF film inside. and (b) high-resolution ABF image of Pt NPs@CNF synthesized through rapid radiative heating.

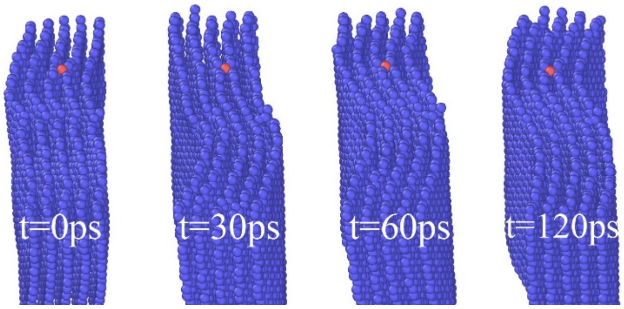

Supplementary Figure 6. The morphological derivation of individual Pt atom in the presence of T-graphene with basal planes

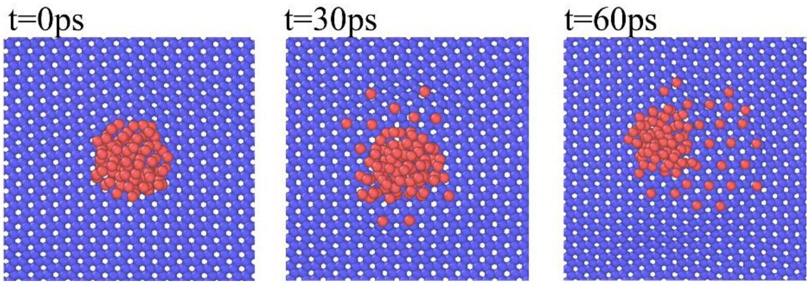

Supplementary Figure 7. The morphological derivation of Pt cluster in the presence of T-graphene basal plane at 1800K.

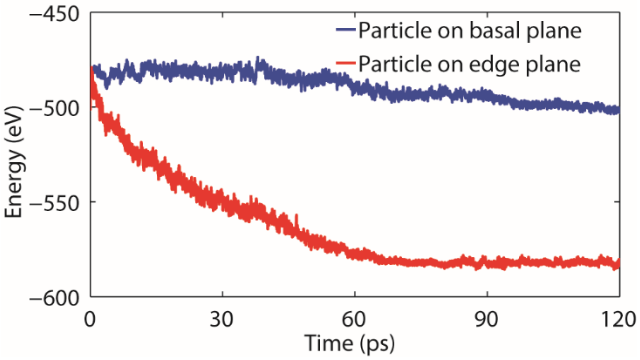

Supplementary Figure 8. Potential energy of the Pt particle as the function of relaxation time in the presence of T-graphene with the basal plane and the edge plane.

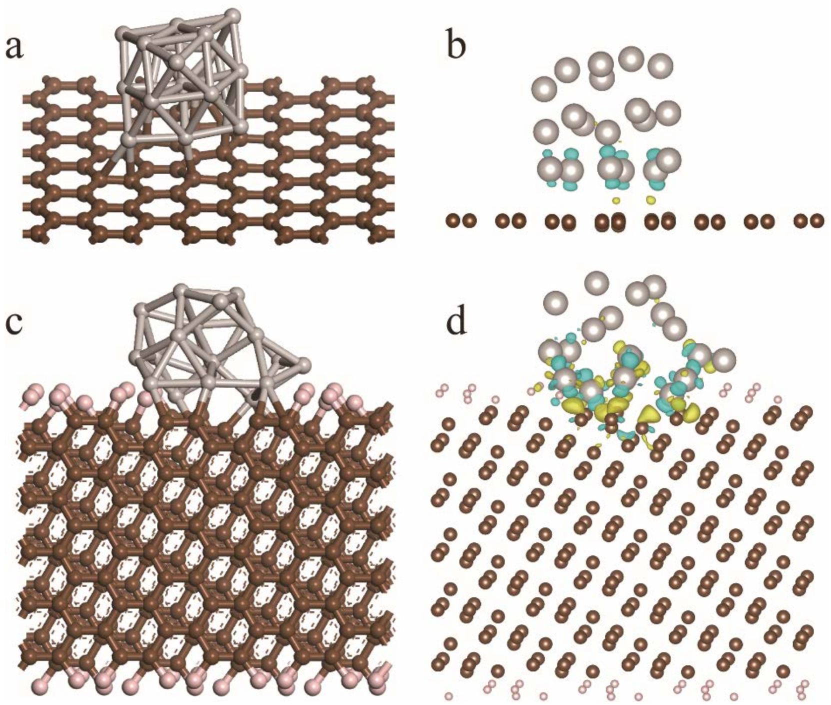

Supplementary Figure 9. (a) Atomistic structure of the Pt particle on the basal plane and (b) the corresponding charge density. (c) Atomistic structure of the Pt particle on the edge plane and (d) the corresponding charge density. Brown, silver and pink balls represent the C, Pt and H atoms, respectively. Yellow and blue regions represent the gain and loss of electrons and the scale level is set as $\pm 0.015e$.

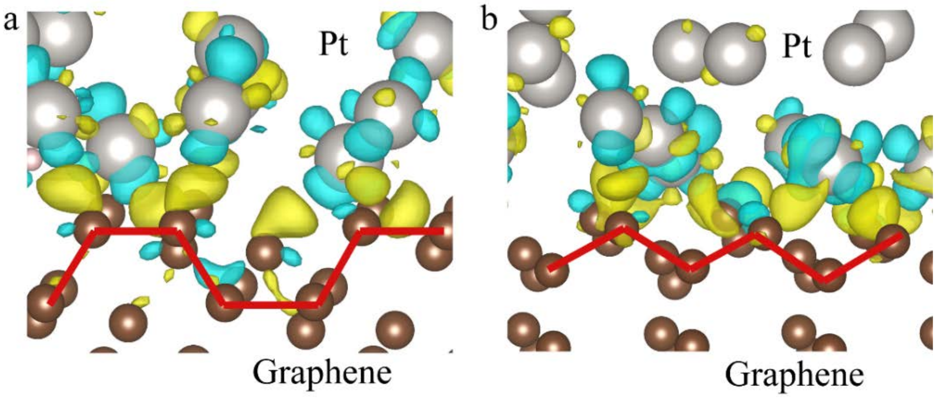

Supplementary Figure 10. Charge density of Pt particle on different types of graphene edges: (a) armchair edge and (b) zigzag edge. The different edges are indicated by red lines. Yellow and blue regions represent the gain and loss of electrons and the scale level is set as ±0.015e.

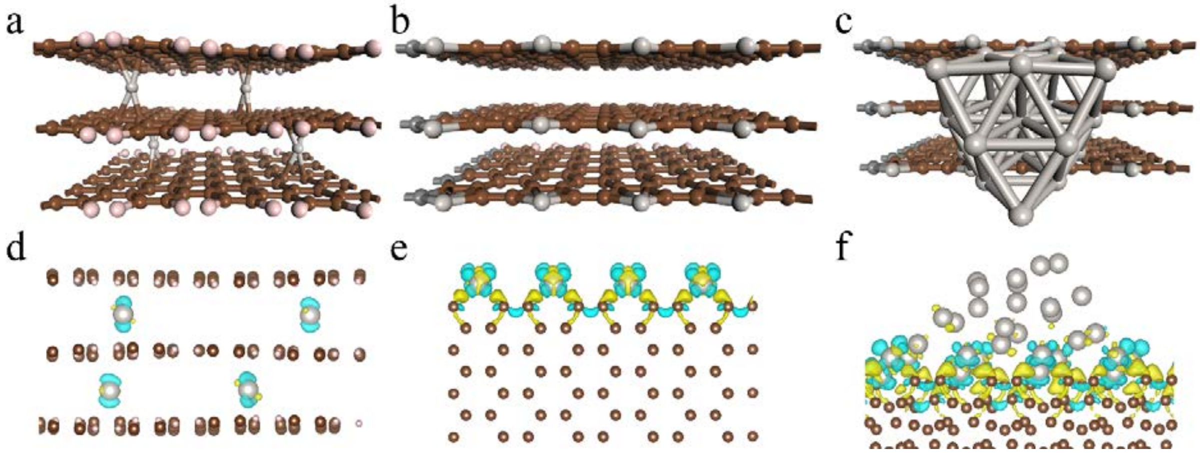

Supplementary Figure 11. Atomistic structure of (a) isolated Pt atoms intercalated graphene, (b) Pt-graphene alloy and (c) Pt particle on the edge plane of Pt-graphene alloy. Brown, silver and pink balls represent the C, Pt and H atoms, respectively. (d-f) Corresponding charge density. Yellow and blue regions represent the gain and loss of electrons and the scale level is set as ±0.015e.

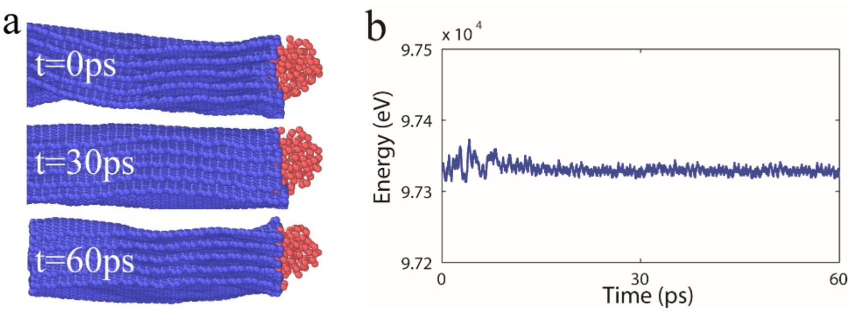

Supplementary Figure 12. (a) The morphological derivation of amorphous Pt cluster in the presence of T-graphene with basal planes at 1000K. (b) Total energy as the function of relaxation time.

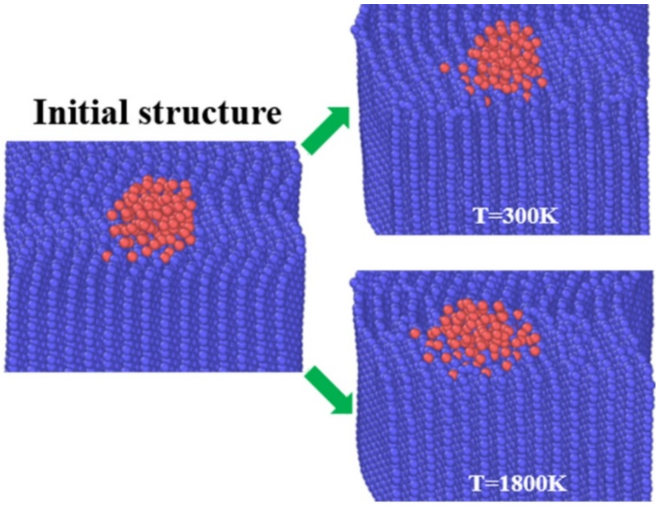

Supplementary Figure 13. The morphological derivation of Pt cluster at 300 K and 1800 K in the presence of T-graphite edge planes

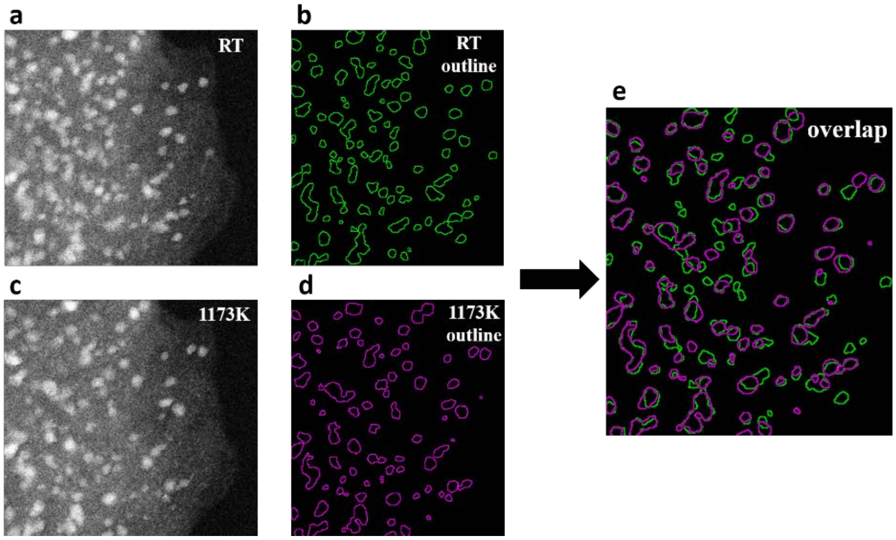

Supplementary Figure 14. Image analysis on the particles at RT and 1173K. (a) The HAADF image of the nanoparticles at RT. (b) The green lines show the border of nanoparticles at RT. (c) The HAADF image of the nanoparticles after 1173K treatment. (d) The purple lines show the border of nanoparticles at 1173K. (e) The overlapped image of the particle to illustrate the movement of nanoparticles by heating from RT to 1173K.

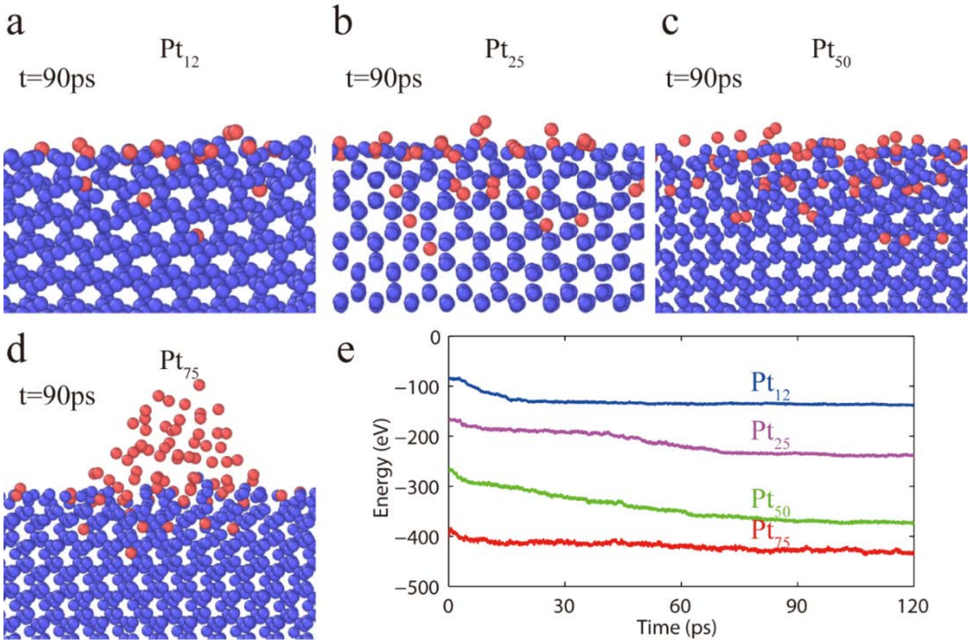

Supplementary Figure 15. The morphological change of Pt particle in the presence of T-graphene with edge plane: (a) Pt_{12}, (b) Pt_{25}, (c) Pt_{50} and (d) Pt_{75}. (e) The potential energy of Pt particles with different sizes as the function of relaxation time.

Supplementary Table 1. A comparison of NPs synthesized through Joule heating and radiative heating methods.

Heating method	Rate	Electric current pass through?	Particle size	CNF substrate
Joule heating shock	Ultrafast(>10^5K/s)	Yes	small	crystalline
Radiative heating shock	Ultrafast(>10^5K/s)	No	small	crystalline
Radiative heating slow	Fast (200K/s)	No	large	amorphous

Supplementary Table 2. The relative motion of particles by comparing the RT and 1173 K treatments.

The status of nanoparticles motion	Percentage
Fixed particles (overlap)	78%
Disappeared particles	16%
Newly appeared particles	4%
Moved particles (not overlap)	3%

Supplementary Table 3. Area and average size of the nanoparticles

Sample	Particle area (nm^2)	Average particle size (nm)
RT	280.5	3.2
1173 K	245.6	3.2

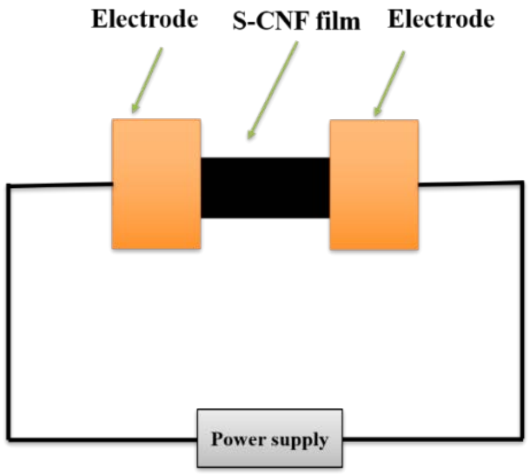

Supplementary Figure 16. Schematic of ex-situ Joule heating setup.

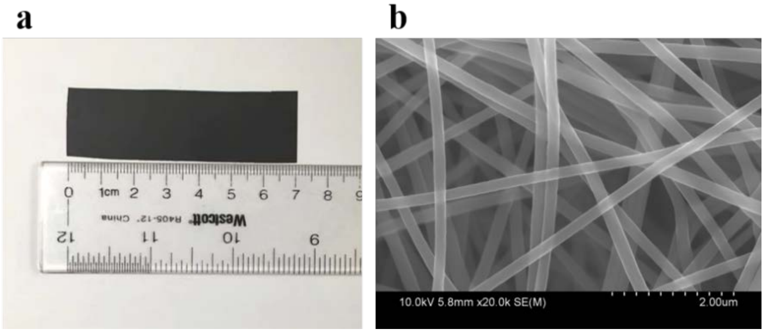

Supplementary Figure 17. Scale-up with a piece of CNF film: (a) millimeter-scale CNF film, and (b) SEM image of the CNF film.

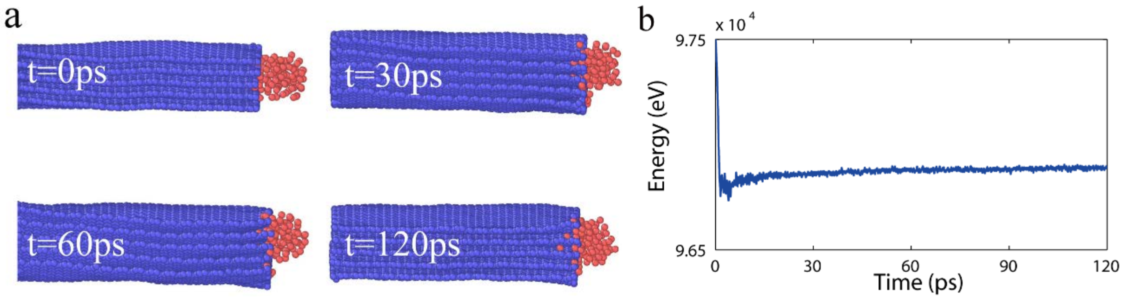

Supplementary Figure 18. (a) The morphological derivation of Pt cluster on the edge plane of T-graphene. (b) Total energy as the function of relaxation time.
