# Supplementary material for: Direct observation of the formation and stabilization of metallic nanoparticles on carbon supports
Source: Nat Commun. 2020 Dec 11;11:6373. doi: 10.1038/s41467-020-20084-5 (PMC7733500; doi:10.1038/s41467-020-20084-5)
Supplement: Supplementary file 3 — Description of Additional Supplementary Files [file 41467_2020_20084_MOESM3_ESM.pdf]

Description of Additional Supplementary Files

File Name: Supplementary Movie 1
Description: In situ Joule heating at low magnification

File Name: Supplementary Movie 2
Description: In situ Joule heating at high magnification

File Name: Supplementary Movie 3
Description: In situ Joule heating captured with 300 fps at low magnification

File Name: Supplementary Movie 4
Description: In situ Joule heating captured with 200 fps at high magnification

File Name: Supplementary Movie 5
Description: In situ radiation heating

File Name: Supplementary Movie 6
Description: Temperature evolution on a CNF through FEA simulation

File Name: Supplementary Movie 7
Description: MD simulation of Pt cluster on five-layer T-graphite edge plane at 1800 K

File Name: Supplementary Movie 8
Description: MD simulation of Pt cluster on fifteen-layer T-graphite edge plane at 300 K

File Name: Supplementary Movie 9
Description: MD simulation of Pt cluster on fifteen-layer T-graphite edge plane at 1800 K
